# Transactional sex among Nigerian university students: The role of family structure and family support

**DOI:** 10.1371/journal.pone.0210349

**Published:** 2019-01-07

**Authors:** Anthony Idowu Ajayi, Oluwaseyi Dolapo Somefun

**Affiliations:** 1 Department of Sociology, University of Fort Hare, East London, South Africa; 2 Demography and Population Studies (DPS), University of the Witwatersrand, Johannesburg, South Africa; University of Westminster, UNITED KINGDOM

## Abstract

**Background:**

The reasons for the persistence of risky sexual behaviours among adolescents and young adults in sub-Saharan Africa despite the increasing knowledge about the associated risks continue to attract scholarly debates. Drawing from a cross-sectional study conducted among male and female Nigerian university students, we examined the relationship between family structure, family support and transactional sex.

**Methods:**

A pre-validated questionnaire was administered to 800 male and female students selected using stratified sampling; however, we performed the analysis on 630 participants who had ever engaged in sex. Transactional sex was operationalised as self-reporting of giving or receiving money, gifts or favour in exchange for sex. We fitted a list-wise logistic regression model to examine the relationship between family structure, family support and transactional sex while controlling for essential covariates.

**Results:**

Of the 630 participants included in the analysis, 17.9% had given and 23.8% had received money, gift or favour in exchange for sex. Our bivariate analysis shows that individuals from polygamous families had higher odds of reporting that they have ever given (AOR: 1.89; CI: 1.05–3.39) or received (AOR: 1.85; CI: 1.85–3.19) money, gift or favour in exchange for sex; however, the relationship was not statistically significant after controlling for relevant covariates. After controlling for essential covariates, the odds of giving or receiving money, gift or favour in exchange for sex was 56% lower in individuals who received adequate family support compared to those who received no or insufficient family support.

**Conclusion:**

In conclusion, this paper lends support to the assertion that family structure and family support are protective factors against transactional sex among adolescents and young adults. Future surveys need to include a larger sample in order to explore the effect of single-parent and polygamous family on transactional sex in Nigeria where family formation is changing rapidly.

## Background

Acquired Immunodeficiency Syndrome (AIDS) is the second leading cause of death among young adults [1]. This makes adolescents and young adults a key priority in the prevention and control of the pandemic nature of the disease. The transition from childhood to adolescence is the most critical phase in the life of an individual. During this phase, adolescents, as a result of peer pressure and other structural factors, are known to engage in risky sexual behaviours [2–5], which could jeopardise their long-term physical and emotional wellbeing [6, 7]. One such risky sexual behaviour is transactional sex, which is incontrovertibly linked with the risk of HIV/STIs transmission [8–10]. Scholars tend to agree that transactional sex is one of the factors fuelling HIV transmission in sub-Saharan Africa [11, 12]–a region with the highest burden of the disease [13, 14]. What is more, available evidence shows that HIV disproportionately affects young people[1]. Of concern is that adolescent girls in sub-Saharan Africa are substantially burdened by HIV, with one in four new HIV infections occurring among this cohort in 2017 [15].

Studies have shown that transactional sex is prevalent among young adults [16–20] in sub-Saharan Africa. Scholars have reported varying levels of transactional sex prevalence among adolescents and young adults in many sub-Saharan Africa settings [20–22]. The reason for this is not only limited to methodological variations adopted in these studies but the fact that there is some ambivalence in defining what constitutes transactional sex. Indeed, there is no consensus among scholars regarding what constitutes transactional sex and how best to operationalize this concept. Although commonly defined as the exchange of money or material gifts for sex [18, 23–25], the exchange of drugs and alcohol for sex was used as a proxy measure of transactional sex in a study [26]. Some scholars only focus on the exchange of sex for money, privileges or favour with men or women who are not regular partners [23], while others focus on both main and casual partners [20, 27]. Others include the initiation of a relationship with a regular partner or staying longer than desired in a relationship because of economic benefits, in their operationalisation of what constitutes transactional sex [25, 28]. Also, the age differences in the relationship have also been used as a proxy measure of transactional sex in some studies [29]. For instance, adolescents or young adults who enter into a relationship with a partner who is over ten years older is said to have entered into a transactional sex relationship [30, 31]. As shown in qualitative studies, young women tend to equate receiving money, gifts or favour in a relationship as a demonstration of love [32, 33]. Thus, the desire to receive a gift is present even in a relationship with peers, which may not be construed as a transactional relationship. The current study defines transactional sex as self-reporting of exchange of sex for money, gifts or privileges with both main and casual partners. Self-reported transactional sex helps to avoid the conundrum of ambiguity in defining what constitutes transactional sex in this study.

Despite the increasing knowledge about the risks associated with transactional sex, the behaviour remains persistent, especially among sub-Saharan African youths. The reasons for why risky sexual behaviours persist among young people continue to attract scholarly debates. Several competing explanations have been advanced to explain this paradox. These competing explanations could be summarised as individual, household and community level factors. At the individual level, some scholars tend to argue that low socioeconomic status of individuals propels them to engage in transactional sex despite the known associated risks [34]. Individual-level factors such as education and early sexual debut are inextricably linked with transactional sex [35]. At the household-level, housing deprivation, food insecurity, and poverty are family level factors reported to influence transactional sex [36–38]. Family structure and family support are also household level factors that could influence transactional sex. However, there is no empirical study to corroborate this assertion. Nonetheless, the fact that the family functions as the basic unit of life and is an essential agent of socialisation in the society supports our proposition. Societal norms and behaviour are learned at the household level and thus influence whether an individual would engage in a transactional relationship. Lastly, place of residence and access to healthcare are the community-level factors that have been found to be associated with transactional sex [38, 39].

The less investigated of these factors are the effects of family structure and family support on transactional sex. Many studies have documented the influence of family structure on youth and adolescents’ outcomes, such as academic achievement, health and well-being [40–42]. Specifically, single-parent and polygamous families are associated with negative child outcomes [43, 44]. We posit that the nuclear family structure and family support could be considered as protective factors against risky sexual behaviour of young people. Some authors [45, 46] have found parental presence to be protective of youth sexual behaviour, but others[47, 48] have gone further to prove that parent-child communication and support could be stronger determining factors for adolescents and young adults’ sexual behaviours. This makes the effect of parental support worth studying as a protective factor against risky sexual behaviour of young people in Nigeria. Drawing from a cross-sectional study conducted among male and female Nigerian university students, we examined the relationship between family structure, family support and transactional sex. Most available studies on transactional sex have focused on young women alone and ignored adolescents and young men. Our study is timely because data on the prevalence of transactional sex in Nigeria is scarce and this information is crucial for designing HIV/AIDS preventative measures mirrored to achieving the sustainable development goal of an HIV-free generation. Also, this study is important considering that Nigeria has the second highest burden of HIV globally [49, 50]. There are over three million people living with HIV in Nigeria and only 30% are on treatment—of which 24% are virally suppressed [49, 50]. In 2016, there were 220,000 new HIV infections in Nigeria and 160,000 AIDS-related deaths [49, 50].

## Theoretical underpinning

We use two frameworks to explain the association between family structure, family support and transactional sex among young adults. We have chosen these theories because they best explain the relationship between family structure, family support and transactional sex among adolescents and young adults. Besides, these theories have not been adequately used to explain transactional sex among youth in the literature. The family structure perspective argues that it is the number and roles of parents that make a difference in children's outcomes. Single parents may have more difficulty in supervising the behaviour of young adults compared to their counterparts who have two parents. It has been documented both in developed [51–53] and developing settings [54, 55] that young adults raised in single-parent households are more likely to exhibit adverse behavioural outcomes. The mechanism through which this occurs could be through the support and parental interaction that could be available in a two-parent household. For instance, research on adolescents in Johannesburg and Baltimore revealed that adolescents perceived the lack of parental presence as a lack of support and guidance [56]. It is also possible that the presence of two parents in the household provides some accountability for each parent to act in desired ways that support the young adult—the advantages of having two parents could be as a result of support from one of the parent if the other is at work. There is evidence that young adults from two-parent households may have more family support available to them which may reduce risky sexual behaviours [57].

Apart from single parenting, coming from a polygamous home could also be a source of stress and instability for young adults in the household [58]. The competition for limited resources in polygamous homes [59] could encourage youths to turn to transactional sex as an alternative means of meeting their needs. Muslims in Nigeria commonly practice polygamy because Islam allows a man to take up to four wives. It is therefore essential to examine how this type of family structure influences transactional sex among males and females in the household.

Economic Deprivation Perspective complements the family structure perspective and explains the issue of family support [60]. Economic Deprivation Theory has been used to explain other youth behavioural outcomes [61], but its association with transactional sex is scarce. Although measured at the community level in some studies [62, 63], the central tenet of this theory is that poverty is associated with adverse behavioural outcomes. Based on this perspective, we deduce that youth who received no or insufficient support from home may engage in transactional sex to sustain their livelihood. We suggest that financial hardship in the household may be as a result of lone parenting or as a result of the death of one of the parent or divorce. Although some authors have argued that it is the socio-economic status of the lone parent that matters, single mothers are still stigmatised in Nigeria and widows may not have access to properties of late husbands in Nigeria, which may influence their status.

Financial difficulties in the household may thus influence the relationship with youth as the lone parent may focus more on immediate needs and ignore other support activities that may influence youth’s positive development [64]. Studies have also established that young adults from financially deprived households may be encouraged by their parents to engage in transactional sex [11, 60].

### Hypotheses

Based on the theoretical background, we posit three hypotheses. The first hypothesis deals with the relationship between family structure and transactional sex. Polygamous home could be a source of stress and instability for young adults in the household [58]. The competition for limited resources in polygamous homes [59] could encourage youths to turn to transactional sex as an alternative means of meeting their needs. Using this reasoning, we expect the polygamous family to be associated with a higher likelihood of engaging in transactional sex. We formally expressed this hypothesis as follows:

**Hypothesis 1:** Polygamous family structure is associated with a higher likelihood of engaging in transactional sex among university students.

The presence of both parents in the household may be advantageous as they could pool resources to support the home and also communicate better with youth in the household. Youth from lone parent households may not have adequate financial support. Also, children from a single parent household may be less supervised compared to their counterparts from a two parents household. This argument leads us to propose the hypothesis on the relationship between living with either parent and engaging in transactional sex:

**Hypothesis 2**: Living with mother alone or living with father alone will be associated with a higher likelihood of engaging in transactional sex.

The Economic Deprivation Theory posits that experience of lack is associated with adverse outcomes in youths. Children from single-parent households or polygamous households may experience some form of deprivation. In other words, the support young adults from nuclear families receive from home may be more compared to their counterpart from single parent or polygamous family. Lack of support from home may lead young adults to engage in transactional sex in order to sustain their livelihood. This reasoning made us posit this hypothesis:

**Hypothesis 3**: Lack of family support is associated with a higher likelihood of engaging in transactional sex among university students.

## Methods

### Study design

We conducted a cross-sectional survey among male and female students in the University of Ilorin (a federal university) and Nasarawa State University (a state-owned university), in North Central Nigeria. Trained research assistants between February and April 2018 administered a pre-validated questionnaire to 800 students. This study was part of a more extensive study which examined the prevalence of HIV testing and awareness of pre-exposure prophylaxis among Nigerian university students. The detailed methodology has been published elsewhere [65]. We based our sample size estimation on the 50% prevalence of HIV testing reported by a previous study [66]. For representativeness, the required sample size per university was 384, at a confidence interval of ±5, and a confidence level of 95%. However, 400 participants were recruited from each university to account for possible incomplete or missing responses. For inclusiveness, participants were selected using stratified random sampling. Respondents were stratified by sex, year of study and faculty of study. We recruited a random sample of eligible participants proportional to the size of each stratum. Also, we removed participants who have never engaged in sex from analysis. Thus, the analysis was limited to only 630 sexually active participants in both universities. The University of Fort Hare and Ondo State Ministry of Health, Nigeria, ethical review bodies granted ethical approval. Each participant provided written informed consent before participation in the study. We provided participants with information detailing the purpose and process of the study. Confidentiality and privacy of the participants were maintained in accordance with the Helsinki Declaration.

### Study measures

#### Dependent variable

The main dependent variable is transactional sex, which was explored with the following questions: “Have you ever given out money, gifts or favours in exchange for sex? “Have you ever received money, gifts or favours in exchange for sex? We provided a dichotomous response (Yes/No) for participants to choose. Transactional sex is defined in this study has an exchange of money, gift or favour for sex.

#### Independent variables

*Family structure* was measured by asking participants to describe their family type and mutually exclusive list (single parent, nuclear family, polygamous family, and foster family) was provided from which they can choose. Also, we asked participants if their fathers are alive and whether they currently live with their father. Similarly, we asked if their mothers are alive and if they live with their mothers.

*Family support* was operationalised in this study by asking participants to self-rate the support they received from their family. We provide a list of mutually exclusive responses (I receive adequate support from my family, I receive moderate support from my family, I receive insufficient support from my family, and I receive no support from my family) from which participants could pick one.

#### Control variables

We controlled for two sets of individual-level variables, which are demographic factors and lifestyle behaviours. Our controls for demographic factors include age and sex. Sex was coded as male and female, while age was categorised into 15–19 years, 20–24 years and 25–34 years. Studies have shown that lifestyle behaviours like alcohol consumption and psychoactive drug are associated with risky sexual behaviour. Thus, we included alcohol consumption and psychoactive drug use as measures of lifestyle behaviours. Alcohol consumption was measured by asking respondents the question, “Do you currently drink alcohol”. We provided a dichotomous response (Yes/No) for participants to choose from. Psychoactive drug use was assessed using the questions: “Do you currently use psychoactive drugs such as codeine, marijuana, tramadol, and crack for pleasure or to ease tension? We coded the responses as Yes (1) or No [67].

### Statistical analysis

We performed analysis on only 630 sexually active participants (data in S1 File). Descriptive statistics were used to describe the demographic characteristics of study participants and the level of transactional sex among Nigerian students. We fitted a list-wise logistic regression model to examine the relationship between family structure, family support and transactional sex while controlling for other relevant variables. Two models with different combinations of family structure, family support, demographic characteristics, drug use, and alcohol use were fitted to examine more succinctly the relationships. **Model 1** is a bivariate logistic regression model examining the independent associations between family structure, family support and the self-reported transactional sex. **Model 2** is a Multivariate logistic regression analysis controlling for demographic characteristics, drug use, alcohol use, and lifestyle behaviours like drug and alcohol use while adjusting for smoking, which is a possible confounder. Interpretations of results were made using odds ratios (OR) with OR > 1 indicating a higher risk, OR < 1 indicating a lower risk and OR = 1 showing no risk difference. The level of significance was set at 0.05, and a confidence interval (CI) of 95% was used. The statistical analysis was done using the Statistical Package for Social Sciences (SPSS version 24.0).

## Results

The mean age of study participants was 22.33 (SD3.28). The demographic characteristics of the study participants are presented in Table 1. Most participants were below 25 years (73.7%), resided off campus, live with at least one roommate (67.3%), from a nuclear family (55.7%), and receives adequate support from home (67.9%). A few participants were active alcohol (33.7%), tobacco (17.8%) and drug users (21.0%). Slightly over half of the sexually active students have ever tested for HIV.

**Table 1:** Demographic characteristics of sexually experienced participants.

Variables	Frequency (N = 630)	Percentage
Sex		
Male	329	52.2
Female	301	47.8
Age Category		
Less than 20	126	20.0
20–24	338	53.7
Above 24	166	26.3
Years in the university		
First	163	25.9
Second	153	24.3
Third	126	20.0
Fourth	146	23.1
Fifth	28	4.4
Sixth	14	2.2
Residence		
Campus residence	114	18.1
Off-campus residence	516	81.9
Living arrangement on campus		
Live alone	206	32.7
Living with one person	247	39.2
Living with more than one person	177	28.1
Family structure		
Single parent	167	26.5
Nuclear	351	55.7
Polygamous family	78	12.4
Foster parent	34	5.4
Family support		
Adequate	428	67.9
Moderate	142	22.5
Insufficient support	42	6.7
No support	18	2.9
Father alive	516	81.9
Living with father	437	69.4
Mother alive	556	88.3
Living with mother	506	80.3
Current alcohol users	212	33.7
Current smokers	112	17.8
Current drug users	132	21.0
Ever tested for HIV	349	55.4

### Descriptive findings

**Fig 1:**
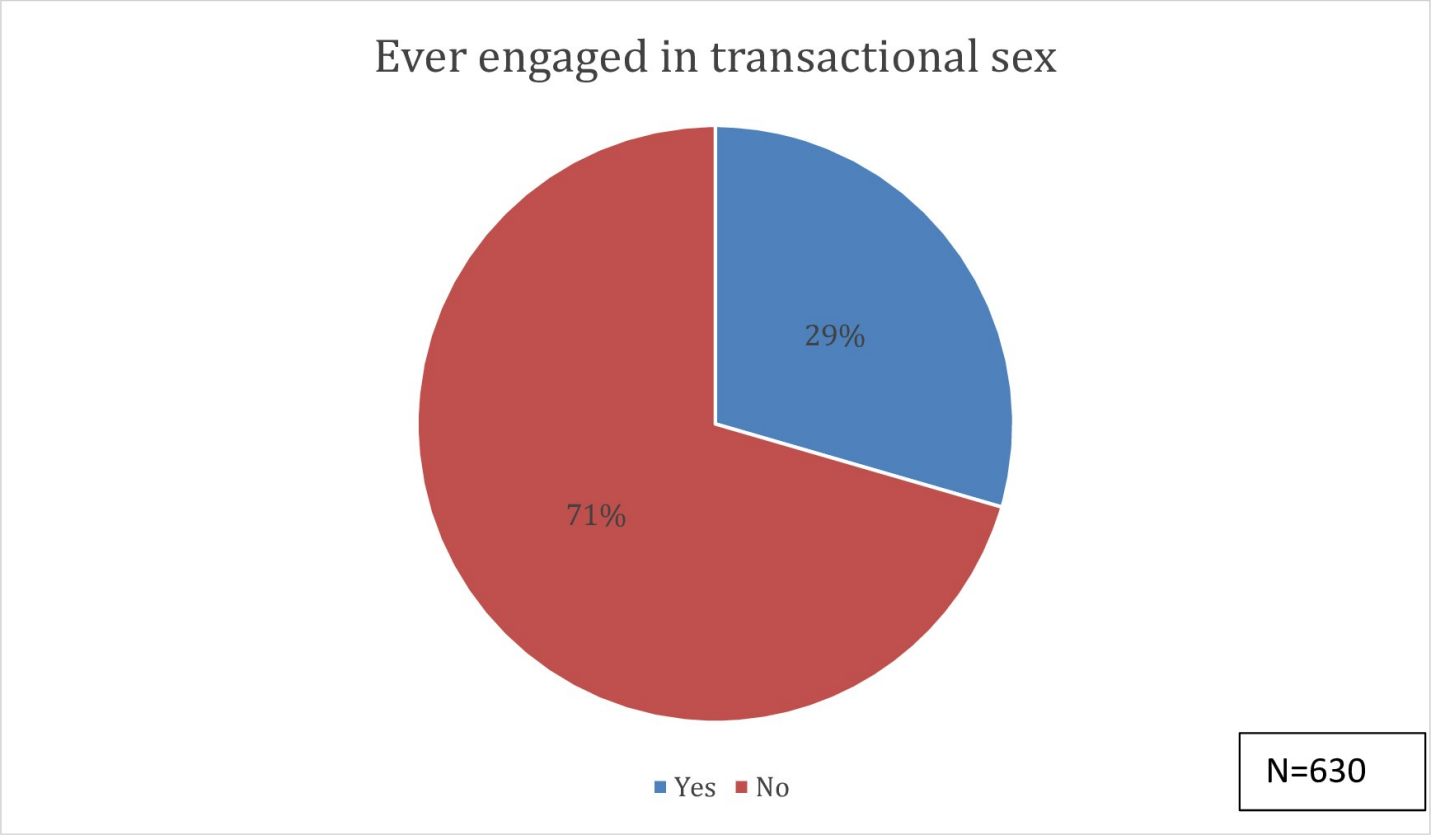
Transactional sex prevalence among Nigerian university students.

The overall prevalence of transactional sex (defined as receiving or giving money, gift or favour in exchange for sex) was 29.2% with significant sex differences (Fig 1). However, only 17.9% of study participants have ever given out money/gift/favour in exchange for sex. There was a significant difference in the proportion of male and female students (24.6% of males versus 10.7% of female) who had ever given out money, gift, or favour in exchange for sex. In contrast, over a fifth (23.8%) of study subjects have ever received gifts, money or favour in exchange for sex. Surprisingly, there was no significant difference in the proportion of male and female students (23.7% of males versus 24% of females) who had ever received money, gift, favour in exchange for sex.

### Multivariate findings

#### Findings on Hypothesis 1: Relationship between family structure and transactional sex

To examine the relationship between family structure and transactional sex, two Binary Logistic Regression Models were fitted and the findings are presented in Table 2. The first model is the baseline model containing no covariates and was used to examine the net effect of family structure on giving and receiving money, gift or favour in exchange for sex. The evidence presented in model provides support for the hypothesis that polygamous family structure is associated with a higher likelihood of engaging in transactional sex. The relationship between family structure and transactional sex is positive (because the estimated odds ratio is greater than 1). Individuals from a polygamous family were about twice as likely to engage in transactional sex compared to individuals from a nuclear family. However, the evidence for this finding did not persist after controlling for demographics and lifestyle behaviours controls. The direction of the relationship remains the same as shown in Model 2, but the confidence interval for odds ratios extend beyond 1. Overall, the evidence of the relationship between family structure and transactional sex is weak, and thus the finding supports the family structure perspective that argues that it is the number and roles of parents that make a difference in children's outcomes rather than the structure of the family itself.

**Table 2:** Binary regression models showing relationship between family structure and transactional sex.

Variables	Giving money in exchange for sex		Receiving money in exchange for sex	
	Model 1 OR (CI)	Model 2 OR (CI)	Model 1 OR (CI)	Model 2 OR (CI)
**Family structure**				
Single parent	1.45 (0.91–2.33)	1.10 (0.65–1.87)	1.45 (0.94–2.23)	1.29 (0.82–2.06)
Polygamous	1.89 (1.05–3.39)*	1.47 (0.77–2.83)	1.85 (1.08–3.19)*	1.50 (0.83–2.70)
Foster family	0.73(0.25–2.16)	0.45 (0.14–1.45)	1.42 (0.63–3.17)	1.03 (0.44–2.45)
Nuclear family (ref)				
Sex				
Male		2.13 (1.31–3.46)*		0.69 (0.46–1.04)
Female (ref)				
Age				
15–19 years		0.40 (0.19–0.85)*		0.51 (0.27–0.96)*
20–24 years		0.65 (0.39–1.06)		0.84 (0.53–1.32)
25–34 years (ref)				
Alcohol use				
Yes		2.66 (1.59–4.44)***		2.07 (1.31–3.25)*
No (ref)				
Drug use				
Yes		3.30 (1.95–5.59)***		3.14 (1.93–5.11)***
No (ref)				

***P-value <0.001

*P-value<0.05

ref-reference

#### Findings on hypothesis 2: Relationship between living in the same household as one’s father or mother and transactional sex

While some students may indicate that they are from a nuclear family, there is a possibility that they may not be living with either of their parents. As argued by the family structure perspective, it is the roles of the parents that make a difference in children’s outcome. Thus, an individual whose either of his/her parent is not present perhaps due to death or divorce may suffer from the vacuum left by the parent. We thus hypothesise that living without either of one’s parent is associated with a higher likelihood of engaging in transactional sex. As shown in Table 3, there is no evidence in support of our hypothesis on the relationship between living in the same household as one’s father and giving money, gift or favour in exchange for sex. However, there is some evidence that living with one’s father reduces the odds for receiving money in exchange for sex. After controlling for demographic and lifestyle behaviours covariates, there was still a negative association between living in the same household as one’s father and engaging in transactional sex, however, the confidence for the odds ratio extend beyond 1, implying lack of support for our hypothesis. The reduction in the magnitude of effect after controlling for covariates suggests that other covariates are essential in explaining transactional sex among the study participants.

**Table 3:** Results of Binary Logistic Regression Models showing asociation between living in the same household as one’s father and transactional sex.

Variables	Giving money in exchange for sex		Receiving money in exchange for sex	
	Model 1 OR (CI)	Model 2 OR (CI)	Model 1 OR (CI)	Model 2 OR (CI)
**Live with father**				
Yes	1.04 (0.66–1.61)	1.42 (0.87–2.33)	0.65 (0.44–0.95)*	0.73 (0.48–1.11)
No (ref)				
Sex				
Male		2.16 (1.33–3.49)*		0.70 (0.46–1.04)
Female (ref)				
Age				
15–19 years		0.39 (0.19–0.83)*		0.51 (0.27–0.95)*
20–24 years		0.64 (0.39–1.05)		0.86 (0.55–1.35)
25–34 years (ref)				
Alcohol use				
Yes		2.75 (1.65–4.57)***		2.11 (1.35–3.31)*
No (ref)				
Drug use				
Yes		3.32 (1.97–5.60)***		3.09 (1.91–5.02)***
No (ref)				

***P-value <0.001

*P-value<0.05

ref-reference

However, there was enough evidence in support of our hypothesis that living with one’s mother is negatively associated with engaging in transactional sex (Table 4). For instance, the odds for giving money, gift or favour in exchange for sex was 47% lower for individuals living with their mothers compared those who do not. Also, the odds for receiving money, gift or favour in exchange for sex is lower among students who live in the same household as their mother relative to those who do not. The direction of the relationship remains the same after controlling for important covariates as shown in Model 2. The evidence we present in Models 1 and 2 provide clear and consistent support for the argument underpinning the role of a mother in our hypothesis.

**Table 4:** Binary Logistic Regression Models showing the relationship between living in the same household as one’s mother and transactional sex.

Variables	Giving money in exchange for sex		Receiving money in exchange for sex	
	Model 1 OR (CI)	Model 2 OR (CI)	Model 1 OR (CI)	Model 2 OR (CI)
**Live with mother**				
Yes	0.34 (0.21–0.54)***	0.53 (0.32–0.90)*	0.45 (0.29–0.69)***	0.62 (0.38–1.00)
No (ref)				
Sex				
Male		2.19 (1.34–3.59)*		0.73 (0.48–1.10)
Female (ref)				
Age				
15–19 years		0.40 (0.18–0.86)*		0.53 (0.28–1.01)
20–24 years		0.65 (0.39–1.07)		0.87 (0.55–1.38)
25–34 years (ref)				
Alcohol use				
Yes		2.54 (1.51–4.29)***		2.02 (1.28–3.21)*
No (ref)				
Drug use				
Yes		3.19 (1.86–5.47)***		3.03 (1.85–4.98)***
No (ref)				

***P-value <0.001

*P-value<0.05

ref-reference.

#### Findings on Hypothesis 3: Relationship between family support and transactional sex

To test the Hypothesis, which deals with the relationship between family support and self-reported transactional sex, we fitted two Binary Logistic regression models. Model 1 is the baseline Model, a null model that contains no covariates. This model was used to establish the net effect of the family support on self-report of giving money, gift or favour in exchange for sex and receiving money, gift or favour in exchange for sex. The findings are displayed in Table 5. Results of Model 1 suggest that the relationship between family support and self-reported transactional sex (defined as giving out favour, gift, and money in exchange for sex) is negative (because the confidence interval of the estimated odds ratio does not extend to 1), implying tentative support for the Hypothesis 4. The evidence for this finding persists after controlling for demographic variables and lifestyle behaviours covariates as shown in Model 2. Results from Model 2 show that the net of all demographic variables and lifestyle behaviours controls, when an individual receives adequate support from home, they are less likely to engage in transactional sex. For example, the odds of giving or receiving money, gift or favour for sex was 56% (1–0.44) lower in individuals who receive adequate support from their parents compared to those who receive no or insufficient support. Although the magnitude of the effect reduces after controlling for relevant covariates, the direction of the relationship remains the same, and the effect size remains very strong. The evidence presented in Model 1 and 2 provide clear and consistent support for the arguments underpinning the economic deprivation theory.

**Table 5:** Binary regression models showing relationship between family support and transactional sex.

Variables	Giving money in exchange for sex		Receiving money in exchange for sex	
	Model 1 OR (CI)	Model 2 OR (CI)	Model 1 OR (CI)	Model 2 OR (CI)
Family support				
Adequate family support	0.26 (0.14–0.47)***	0.44 (0.22–0.85)*	0.27 (0.16–0.48)***	0.44 (0.24–0.82)*
Moderate family support	0.45 (0.23–0.87)*	0.56 (0.27–1.17)	0.34 (0.18–0.64)*	0.45 (0.23–0.90)*
No/insufficient support (ref)				
Sex				
Male		2.24 (1.38–3.64)*		0.72 (0.48–1.09)
Female (ref)				
Age				
15–19 years		0.48 (0.23–1.03)		0.56 (0.29–1.06)
20–24 years		0.71 (0.43–1.17)		0.86 (0.55–1.37)
25–34 years (ref)				
Alcohol use				
Yes		2.63 (1.58–4.38)***		2.01 (1.31–3.25)***
No (ref)				
Drug use				
Yes		2.93 (1.73–4.97)***		2.90 (1.77–4.74)***
No (ref)				

***P-value <0.001

*P-value<0.05

ref-reference

## Discussion

A paucity of literature on the effect of family structure and family support on transactional sex motivates this study. While there are studies on how single-parent family structure leads to adverse outcomes for adolescents, literature focusing on behavioural consequences for children such as transactional sex is still developing. We hypothesise in this paper that individuals from a polygamous family structure have a higher likelihood of engaging in transactional sex compared to those from a nuclear family. The finding of this study shows that the association between polygamous family structure and transactional sex is positive. Our interpretation of the results is that polygamous family sizes are larger than that of a nuclear family, implying that there is a considerable constraint on resources available to children from such families.

Consequently, individuals from such families may relatively be more deprived of resources to meet their needs. These resources could be in form of finance or time allocated to communicate with the youth in the household. Studies have shown that material, housing and food deprivation are associated with transactional sex [36–38]. Besides this, polygamous family is characterised by parental conflicts and other family dysfunctions, which are detrimental to the well-being of a child [40–42]. The tumultuous environment in a polygamous family may not be ideal for child upbringing and could negatively affect the outcome of the child. Unlike the polygamous family, the nuclear family may offer a more protective and cordial environment for young adults to develop positive traits. Even though we found evidence in support of our hypothesis, the confidence intervals for the odds ratios reported in our findings suggest that our hypothesis may not be true if the entire population of young adults are studied. There is, therefore, a need to for further studies to explore the association between family structure and transactional sex.

While we do not find evidence in support of our argument that living in the same household as one’s father is negatively associated with transactional sex, our findings clearly show that living in the same household as one’s mother reduces the odds for engaging in transactional sex. Our interpretation of this finding is based on the family structure perspective that argues that it is the number and roles of parents that make a difference in children's outcomes. Adolescents and young adults who do not live with their parents, either as a result of death or divorce, may be more deprived of motherly attention and care compared to their counterparts who do. Mothers are the primary source of support for adolescents and young adults. Once this pillar of support is removed either by death or divorce, adolescents and young adults may have to fend for themselves with transactional sex being an accessible option for meeting their needs [36, 68]. Studies have shown that the death of a mother can devastate the health and economic well-being of a family [69–72]. The death of a mother not only represents an economic loss to the family, but it also denotes the loss of the primary caregiver of the family [69–72]. There is evidence that children whose mother died are abandoned by their fathers, undernourished, forced to drop out of school, to take on difficult household and farm tasks and are far less likely to survive [69–72]. Our study further shows that the absence of a mother is detrimental to a child and transactional sex may be one coping mechanism adolescents and young adults embrace to deal with this loss.

The dearth of literature on the role of family support in mitigating transactional sex also motivates this study. In previous pages, we argued that the relationship between family support and transactional sex is negative. The findings of this study reveal a robust negative association between family support and transactional sex. Our interpretation of the results is that lack of family support makes adolescents and young adults more vulnerable to transactional sex. The interpretation is consistent with the argument of the economic deprivation perspective and extant literature, which assert that the experience of food, material and housing deprivation are risk factors for transactional sex among young adults [36–38]. The campus environment offers a high degree of freedom to adolescents and young adults, who are probably living on their own for the first time and without their parents’ guidance. The need to be socially compatible with peers, which entails dressing in a particular fashionable way and using the latest smartphones, has been reported to be associated with transactional sex [36–38]. Individuals who receive no support or insufficient support from home may not be able to afford their desired campus lifestyle without engaging in transactional sex.

The findings of this study have important implications for sexuality studies and public health policies. In Nigeria universities, unlike in the developed countries, little or no support is available for indigent students on campuses. Considering the broad societal implications of transactional sex on adolescents and young adults, providing funding opportunities for indigent students could be a timely intervention in the study settings. The current neglect of vulnerable adolescents and young adults on Nigeria campuses is no longer tolerable.

### Study limitations

Even though this study contributes to the extant literature on transactional sex, the findings should be situated within its limitations. First, the use of a cross-sectional design in this study indicates that the association between family structure, family support and transactional sex do not infer causation. Also, the importance of social desirability bias in self-reported sexual behaviours, in this case giving and receiving money, gift or favour for sex, may lead to under-reporting of transactional sex prevalence. Also, this study was conducted among Nigeria university students who generally are more educated than average adolescents and young adults’ population thus; the findings are not generalizable to the overall Nigerian adolescents and young adults’ population.

## Conclusion

In conclusion, this paper lends support to the assertion that family structure and family support are protective factors against transactional sex among adolescents and young adults. Future surveys need to include a larger sample in order to explore the effect of single-parent and polygamous family on transactional sex further.

## Supporting information

S1 File Dataset- sexually active only. (SAV)

## Abbreviations

AIDS: acquired immunodeficiency syndrome
HIV: Human immunodeficiency virus
STIs: sexually transmitted infections
